# A better alignment between chronotype and school timing is associated with lower grade retention in adolescents

**DOI:** 10.1038/s41539-023-00171-0

**Published:** 2023-06-21

**Authors:** Guadalupe Rodríguez Ferrante, Andrea P. Goldin, Mariano Sigman, María Juliana Leone

**Affiliations:** 1 Universidad Torcuato Di Tella, CONICET, Laboratorio de Neurociencia, C1428BIJ Buenos Aires, Argentina; 2 Universidad Nacional de Quilmes, CONICET, Laboratorio de Cronobiología, Departamento de Ciencia y Tecnología, B1876BXD Bernal, Buenos Aires Argentina; 3 Facultad de Lenguas y Educación, Universidad Nebrija, Madrid, Spain; 4 Universidad Torcuato Di Tella, CONICET, Área de Educación, Escuela de Gobierno, C1428BIJ Buenos Aires, Argentina

**Keywords:** Neuroscience, Psychology

## Abstract

Schools start early in the morning all over the world, contrasting with adolescents’ late chronotype. Interestingly, lower academic performance (i.e. grades or qualifications) was associated with later chronotypes. However, it is unclear whether it is a direct effect of chronotype or because students attend school too early to perform at their best. Moreover, little is known about how this affects students’ academic success beyond their grades. To address this gap in knowledge, we studied how school timing and chronotype affect grade retention (i.e. repeat a year) in a unique sample of students randomly assigned to one of three different school timings (starting at 07:45, 12:40, or 17:20). Even when controlling for academic performance, we found that later chronotypes exhibit higher odds of grade retention only in the morning, but not in later school timings. Altogether, ensuring a better alignment between school timing and students’ biological rhythms might enhance future opportunities of adolescents.

## Introduction

Humans exhibit circadian or close to 24 h rhythms in their physiology and behavior, which differ between individuals. These differences are captured by chronotype, which is the expression of each individuals’ endogenous circadian timing under specific conditions (including the light-dark cycle)^1,2^, and ranges on a continuum between early and late types. Chronotype has a genetic basis^3–5^, but it is also modulated by multiple factors, such as light exposure^1,6^, age^7,8^ and cultural and social cues^6,9,10^. It can be assessed by evaluating behavioral^11–13^ and/or physiological rhythms^14–16^ as well as with standardized questionnaires. One of the most widely used questionnaires is the Munich chronotype questionnaire (MCTQ)^17^, from which the sleep-corrected Midpoint of Sleep on Free days (MSFsc) is obtained. MSFsc is a chronotype proxy based on sleep timing and it highly correlates^18,19^ with the phase of sleep-rest activity^11–13,17,20^ (evaluated with sleep diaries and actigraphy) and endogenous physiological rhythms^14–16,21–23^. Thus, this questionnaire is an easy and reliable method to assess chronotype.

Secondary school starts very early in the morning for most students around the globe. Students´ chronotypes at this age become progressively delayed reaching a peak of lateness at the end of adolescence^7,8,24^. This misalignment between biological timing (i.e. chronotype) and social obligations (i.e. school schedule) is proposed to be the main cause why many adolescents present chronic sleep deprivation and social jetlag (i.e. discrepancy of sleep timing between free days and weekdays), which in turn, show to be associated with health problems and impaired cognitive performance^25–31^. Several interventions delaying school start time lead to an improvement of adolescents’ mood, wellbeing^32–34^ and academic performance^35,36^. Although not conclusive^37^, these results suggest that a better alignment between adolescents’ internal timing and school schedules could be beneficial to improve adolescents’ academic performance.

Although adolescents exhibit later chronotypes than children and adults, there is a large intrinsic variability in their chronotypes^7,8^. Some studies show that students with earlier chronotypes attending school in the morning perform better than their peers with later chronotypes^38–40^. However, it is not clear whether this result occurs because early chronotypes perform better than late chronotypes (‘chronotype effect’) or because early chronotypes, unlike late chronotypes, are being evaluated at their best time of the day (‘synchrony effect’). Evidence of the synchrony effect was found in executive functioning^41–43^, priming^44^, memory^45^, and fluid (but not crystallized) intelligence^46,47^. At school, results vary according to which school subjects are considered. Morning-attending students perform better in math and chemistry if they present an early chronotype. However, this effect is smaller or absent for native language and geography^48,49^. Therefore, the chronotype and/or the synchrony effects might differentially affect performance depending on school subjects. Finally, because chronotype progressively delays during adolescence, younger students are expected to be less affected than older ones.

There are only a handful of studies comparing how academic performance (measured as school grades, hereafter used as synonyms) is affected by chronotype in morning and afternoon school timings. These studies showed that adolescents with earlier chronotypes perform better in the morning school timing, but not in the afternoon, where academic performance does not vary across chronotypes^50–55^. This seems to imply that chronotype is not the only factor affecting adolescents’ academic performance, but this conclusion cannot be established because, in these experiments, students were not randomly assigned to school timings. Thus, results can be bias because students’ preferences and baseline differences in chronotype and academic performance between school timings. In addition, these results are compatible with a pure effect of synchrony which could be masked if the afternoon school timing is yet too early for late chronotypes to perform better than early chronotypes. Hence these studies cannot rule out between these different scenarios explaining how the interaction between chronotype and school timing affect adolescents’ grades: (1) variations in academic performance are completely explained by the interaction between chronotype and school timing, with higher grades associated with a better alignment between school schedules and students’ chronotype (i.e. synchrony effect); (2) variations in academic performance are completely explained by chronotype, with earlier chronotypes obtaining higher grades than later chronotypes (i.e. chronotype effect); (3) both the chronotype and its interaction with school timing modulate academic performance; as a result, earlier chronotypes perform better, but the magnitude of this association will be related to how well chronotype and school timing are aligned (i.e. both chronotype and synchrony effects)^56^.

Recently, in a cross-sectional study of our group we try to disambiguate these possible scenarios addressing the mentioned confounds (lack of random assignment and lack of an evening school timing). We investigated performance in a natural educational setup where students in their first year were randomly assigned to one of three different school timings: morning (07:45–12:05), afternoon (12:40–17:00), or evening (17:20–21:40)^56^. This random assignation suggests no bias for factors that can condition school timing assignment, such as socio-economic status, chronotype preferences or previous academic achievement^51^. The study showed that, for morning-attending students, early chronotypes performed better than late chronotypes in all school subjects and, particularly, in math. On the other hand, this effect was not observed in any school subject for students who attended school in the afternoon. Finally, older students with late chronotypes benefit from evening classes, especially on native language^56^.

Here, we present a longitudinal study, further capitalizing this unique educational setup. We evaluated students in their 1st (13–14 years old) and 5th (17–18 years old) year of secondary school^56,57^. This longitudinal design allows us to assume that the differences observed between 1st and 5th year in students’ academic performance are not due to interindividual variability but due to age-related changes. Moreover, chronotype interacting with school schedule could not only modulate student academic performance (i.e. grades), but also more global measures of school success such as grade retention (i.e., the proportion of students that repeat at least one school year throughout secondary school). In the last few years it was reported that short sleep duration predicts class retention in college students^58,59^, but the effect of chronotype on grade retention in adolescents remains unknown. Our longitudinal study providing data of which students that start their 1st year in 2015 do not reach 5th year four years later, allowed us to address this gap in knowledge.

The aim of this work is to understand whether, and how, chronotype interacting with school timing affects academic success, measured as both academic performance (i.e. school grades) and grade retention (i.e. repeat a year). The impact of chronotype and school timing in academic success was addressed in three different ways. First, we test whether academic performance differs between school timings in 1st and 5th year: a better performance in one of the three school timings could indicate a better alignment between school schedules and students’ internal timing. As Argentinian adolescents’ present particularly late chronotypes^57^, we hypothesize that students attending afternoon and/or evening school timings will present better academic performance than those attending school in the morning (synchrony effect). Second, we study how synchrony and chronotype effects can modulate academic performance considering interindividual differences in adolescents’ chronotype. Taking into consideration our previous cross-sectional results^56^, here we hypothesize that both synchrony and chronotype effects will act together to modulate academic performance. Third and importantly, we study whether chronotype interacting with school timing predicts grade retention. We hypothesize that, even controlling for academic performance, students with later chronotypes would present higher odds of experiencing grade retention (i.e. not reaching their last school year) when attending morning school timing; and that this effect would be gradually lower for later school timings.

Altogether, here we study whether and how chronotype alone and/or including its alignment with the school schedule affect academic success.

## Results

### Academic performance is higher in later school timings (synchrony effect)

To test whether and how academic performance is affected by school timing during adolescence, we ran a linear mixed effect model with academic performance as dependent variable and school timing, age (1st or 5th year), school subject and their interactions as predictors. We included students’ id, students’ classroom and type of grade as random factors (see methods for details). Grade means are in Supp. Table 1 and ANOVA results are in Supp. Table 2. Academic performance was significantly affected by school subject (*F*_1257_ = 503.070, *P* < 0.0001, partial *η*2 = 0.056, 90% confidence interval (CI) = 0.051–0.062) and by its interaction with age (*F*_1257_ = 41.736, *P* < 0.0001, partial *η*2 = 0.005, 90% CI = 0.003–0.007). More importantly, we found that academic performance was significantly affected by school timing interacting with both age and school subject (*F*_1257_ = 17.967, *P* < 0.0001, partial *η*2 = 0.004, 90% CI = 0.003–0.006; post-hoc pairwise comparisons between school timings for each combination of age and school subject is shown in Supp. Fig. 1 and Supp. Table 3). When adolescents were younger (i.e. at their 1st year), we observed better math performance in afternoon-attending students than in their evening-attending peers (*t* = 3.018, *P* = 0.007, Cohen’s *d* = 0.411, 95% CI = [0.144–0.677]). In addition, both afternoon- and evening-attending students tended to present better language (Spanish) performance than their morning-attending peers, however it does not reach significance (morning vs. afternoon: *t* = −2.177, *P* = 0.075, Cohen’s *d* = −0.287, 95% CI = [−0.545 to −0.029]; morning vs. evening: *t* = −2.183, *P* = 0.074, Cohen’s *d* = −0.298, 95% CI = [−0.595 to −0.030]). On the other hand, when adolescents were older (i.e. in their 5th year), evening-attending students presented better math performance than their morning- and afternoon-attending peers (*t* = −3.432, *P* = 0.002, Cohen’s *d* = −0.468, 95% CI = [−0.736 to −0.201]; and *t* = −2.908, *P* = 0.01, Cohen’s *d* = −0.396, 95% CI = [−0.663 to −0.129], respectively), while no differences were found in language.

Altogether, and consistent with our prediction, students in their 1st year perform better (or equal, depending on the school subject) in the afternoon school timing and, when they grow-up (i.e. at their 5th year), they perform better in math later, in the evening school timing. This could be due to the fact that during 1st year students’ internal timing is better aligned with afternoon school timing, while during 5th year, it is better aligned with the evening school timing. This is especially plausible considering that students in first year present earlier chronotypes^57^.

### Academic performance is modulated by chronotype and its interaction with age and school timing (chronotype and synchrony effects)

In this section, we study how interindividual differences in chronotype within and between school timings affect academic performance and for that, we included chronotype (i.e. MSFsc) in the analysis. Specifically, we ran a linear mixed effect model in which we included the interplay between chronotype, age, school timing, and school subject as predictors of academic performance. We controlled for gender in order to avoid a possible cofounding between this variable and MSFsc, as there are reports indicating that gender could influence chronotype^8^. We included students’ id, students’ classroom and type of grade as random factors (ANOVA and summary results are in Supp. Tables 4 and 5 respectively). We observed a main effect of chronotype (*F*_1257_ = 25.264, *P* < 0.0001, partial *η*2 = 0.041, 90% CI = 0.019–0.070), school subject (*F*_1256_ = 2203.130, *P* < 0.0001, partial *η*2 = 0.048, 90% CI = 0.043–0.054) on academic performance. We also observed a main effect of gender (*F*_1257_ = 54.311, *P* < 0.0001, partial *η*2 = 0.074, 90% CI = 0.031–0.129), with girls performing consistently better than boys (Supp. Tab. 1). Although we did not observe a main effect of school timing (*F*_1256_ = 11.812, *P* = 0.110, partial *η*2 = 0.082, 90% CI = 0.000–0.205) or age (*F*_1257_ = 5.810, *P* = 0.149, partial *η*2 = 0.110, 90% CI = 0.000–0.355), both of them do interact with the other factors to explain students’ academic performance (Supp. Table 4). Importantly, school timing interacting with both chronotype and school subject significantly affect academic performance (*F*_1254_ = 56.832, *P* < 0.001, partial *η*2 = 0.001, 90% CI = 0.000–0.002). In addition, academic performance is modulated by chronotype interacting with age (*F*_1257_ = 10.958, *P* = 0.039, partial *η*2 = 0.009, 90% CI = 0.000–0.029), with school subject (*F*_1256_ = 102.361, *P* < 0.001, partial *η*2 = 0.002, 90% CI = 0.001–0.004), and with both factors together (*F*_1256_ = 33.871, *P* = 0.001, partial *η*2 = 0.001, 90% CI = 0.00–0.002). The interaction between the four factors was not significant (*F*_1254_ = 17.213, *P* = 0.152, partial *η*2 = 0.000, 90% CI = 0.000–0.001).

**Fig. 1:**
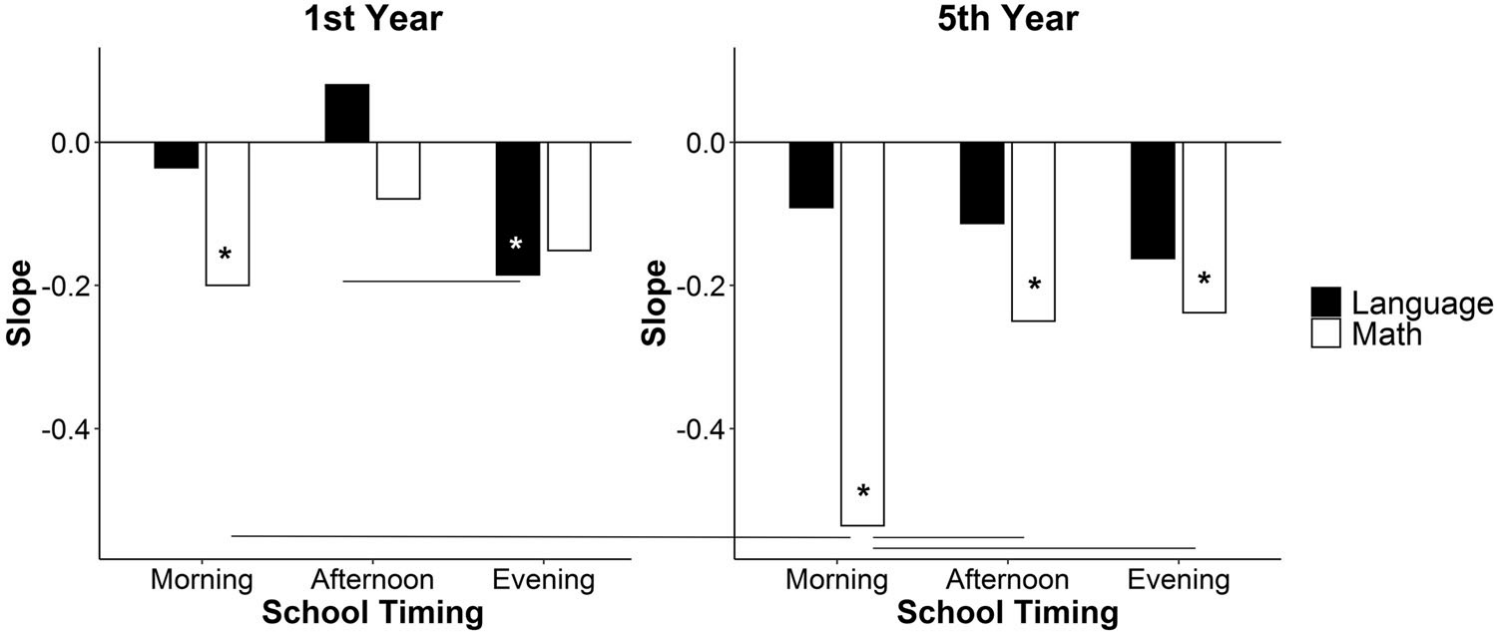
Slopes of the regression lines between chronotype (i.e. MSFsc) and academic performance depend on school timing, age and school subject. Slopes for 1st year and 5th year students. A null slope implies no association. A negative slope indicates higher academic performance for earlier chronotypes. A positive slope indicates higher academic performance for later chronotypes; raw values are provided in Table 1; *n* = 259. The lines indicate the significant pairwise comparisons. Asterisks (*) indicate which slopes differ from zero.

Figure 1 and Table 1 show the slopes describing the relation between academic performance and chronotype for the different combinations of the factor’s levels included in the model (Supp. Figs. 2 and 3 show the regression lines and the data points; Supp. Table 6 shows comparisons between slopes).

**Table 1:** Slopes intercepts and grades for a chronotype 1 h later than average.

Age group	School subject	School timing	Grade for 1 h later MSFsc	Intercept	Slope	95% CI	*z*	*P*-value
1st Year	Other subjects	Morning	7.661	7.655	0.006	[−0.092–0.104]	0.122	0.903
1st Year	Other subjects	Afternoon	7.708	7.801	−0.094	[−0.197–0.010]	−1.766	0.077
1st Year	Other subjects	Evening	7.499	7.604	−0.105	[−0.223–0.012]	−1.756	0.079
5th Year	Other subjects	Morning	7.673	7.707	−0.034	[−0.151–0.084]	−0.564	0.573
5th Year	Other subjects	Afternoon	7.654	7.697	−0.043	[−0.146–0.059]	−0.826	0.409
5th Year	Other subjects	Evening	7.734	7.843	−0.110	[−0.223–0.004]	−1.895	0.058
1st Year	Language	Morning	6.776	6.812	−0.036	[−0.179 to 0.107]	−0.490	0.624
1st Year	Language	Afternoon	7.286	7.206	0.080	[−0.070–0.231]	1.045	0.296
1st Year	Language	Evening	7.145	7.330	−0.185	[−0.354 to −0.017]	−2.153	0.031
5th Year	Language	Morning	7.740	7.831	−0.091	[−0.264–0.081]	−1.037	0.300
5th Year	Language	Afternoon	7.666	7.779	−0.113	[−0.268–0.041]	−1.438	0.150
5th Year	Language	Evening	7.938	8.101	−0.162	[−0.328–0.004]	−1.918	0.055
1st Year	Math	Morning	6.407	6.607	−0.200	[−0.343 to −0.057]	−2.746	0.006
1st Year	Math	Afternoon	6.857	6.936	−0.079	[−0.230–0.071]	−1.030	0.303
1st Year	Math	Evening	6.135	6.286	−0.151	[−0.320–0.017]	−1.757	0.079
5th Year	Math	Morning	5.107	5.642	−0.535	[−0.708 to −0.363]	−6.090	<0.0001
5th Year	Math	Afternoon	6.099	6.349	−0.250	[−0.404 to −0.096]	−3.181	0.002
5th Year	Math	Evening	6.797	7.035	−0.238	[−0.404 to −0.072]	−2.814	0.005

To obtain a more natural interpretation of the model’s estimates, MSFsc was included relative to its global mean (*M* = 06:27). Each intercept results from the sum of the corresponding coefficients and indicates the predicted grade on each group of conditions for a female student with an average chronotype. Each slope indicates the predicted change in grades for an MSFsc of 1 h later. A two-sided *z*-test was performed to test the significance of each slope. *P*-values were computed using lmerTest package^79^. Slopes are considered to significantly differ from 0 when *p*-values are <0.05. *n* = 259.

At their 1st year, students with later chronotypes obtain lower performance than their earlier peers in math when attending school in the morning and in language when attending in the evening (*β* = −0.200, 95% CI = −0.343 to −0.057, *t* = −2.746, *P* = 0.006 and *β* = −0.185, 95% CI = −0.354 to −0.017, *t* = −2.153, *P* = 0.031, respectively). In the afternoon, later chronotypes were not associated with lower academic performance (math: *β* = −0.079, 95% CI = −0.230–0.071, *t* = −1.030, *P* = 0.303; language: *β* = 0.080, 95% CI = −0.070–0.231, *t* = 1.045, *P* = 0.296). Additionally, the magnitude of the association between chronotype and language performance in 1st year is significantly more negative in the evening than in the afternoon (*t* = 2.302, *d* = 0.166, 95% CI = 0.025–0.307).

When students are older, although all slopes are negative, only slopes for math performance differ from zero (morning: *β* = −0.535, 95% CI = −0.708 to −0.363, *t* = −6.09, *P* < 0.0001; afternoon: *β* = −0.250, 95% CI = −0.404 to −0.096, *t* = −3.181, *P* = 0.002; evening: *β* = −0.238, 95% CI = −0.404 to −0.072, *t* = −2.814, *P* = 0.005). This result indicates that the association between later chronotypes and lower academic performance is strong for math in all school timings, suggesting the existence of a chronotype effect. However, the effect is stronger for morning-attending students: while in the morning a 1 h later chronotype is associated with a decrease of 0.535 points on math grades, in the afternoon and the evening the decrease is 0.250 and 0.238 points, respectively (morning vs. afternoon: *t* = −2.421, *d* = −0.178, 95% CI = −0.323 to −0.034; morning vs. evening: *t* = −2.436, *d* = −0.186, 95% CI = −0.335 to −0.036). The latter supports the existence of a synchrony effect that partially counteracts the chronotype effect (i.e. lower academic performance in later chronotypes).

In summary, academic performance seems to be modulated by the chronotype effect, which is stronger for math than for language, especially when students are older. Importantly, this chronotype effect observed on math performance is modulated by a synchrony effect, reducing the impact of chronotype on later school timings.

### Grade retention is predicted by both academic performance and chronotype interacting with school timing (synchrony effect)

There are other relevant outcomes associated with academic success besides academic performance; one of them is grade retention. Here, we hypothesize that the synchrony effect (i.e. the alignment between chronotype and school timing) predicts grade retention. Here we can test this hypothesis because we know chronotype and school timing of 1st year students and, also, which of these students reached their 5th, and last, year of secondary school. Using these data, we ran a set of iterative logistic regression models to test which model was the best to explain grade retention and we chose the most parsimonious under Akaike criterion (Supp. Table 7). Because academic performance is an important predictor of grade retention^60,61^, we included both math and language grades along with chronotype (i.e. chronotype effect) and chronotype interacting with school timing (i.e. synchrony effect) as explanatory variables in the initial model. Then, we successively added other relevant factors and interactions (Supp. Table 7). The most parsimonious model included the initial predictors and the interactions between school timing and language grades and between chronotype and math grades (model 5 in Supp. Table 7).

**Fig. 2:**
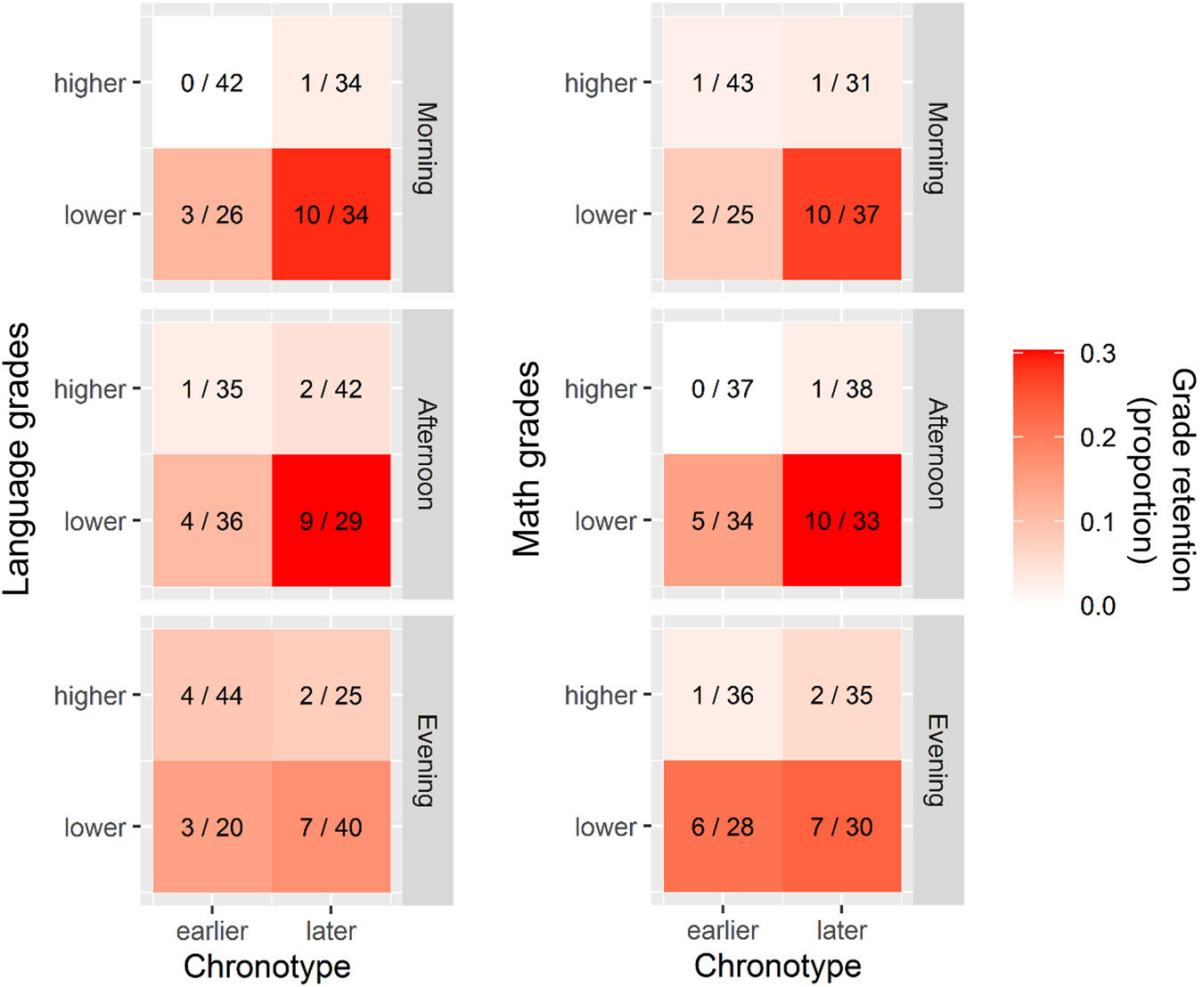
Grade retention depends on academic performance, chronotype, and its interaction with school timing. The proportion of repeating students in different school timings varies between quadrants defined by chronotype (i.e. MSFsc) and grades medians. We calculated the mean grade for either Math and Language for each student, and then we divided those values in two groups in each school timing: the ones who are above the median (higher or equal) and the ones below it (lower). We also obtained two groups in each school timing for individuals’ chronotype: the one with MSFsc values above (later) and the ones with values below (earlier) the MSFsc median. Then, for each school timing and school subject, four quadrants were defined by the intersection of chronotype and grades (either language or math) medians. Then, for each school timing, four quadrants were defined by the intersection of chronotype and language and math grades medians. The number on the right on each quadrant represents the total number of 1st year students that belong to this quadrant and the number on the left represents the quantity of those students that did not timely reach 5th year (i.e. who were not at school four years later). Color represents the ratio between both numbers, which is the proportion of students that did not reach timely 5th year (a darker red indicates a higher proportion of students who repeat a grade). Note that this figure is useful to illustrate the results, but MSFsc and math and language grades were included as numerical variables in the model, even though here we represent them as factors. *n* = 407.

The selected model shows a main effect of both math (*F*_1406_ = 51.156, *P* < 0.0001, partial *η*2 = 0.130, 90% CI = 0.083–0.182) and language grades (*F*_1406_ = 18.006, *P* < 0.0001, partial *η*2 = 0.046, 90% CI = 0.018–0.084), which means that lower 1st year grades are associated with higher grade retention (Fig. 2). Although the interaction between language grades and school timing was not significant, the summary of the model shows a significant difference in the effect of language grades between evening and morning school timings (Evening, Language grades: *β* = 0.694, 95% CI = 0.200–1.277, *t* = 2.585, *P* = 0.010). In addition, the Beta for the interaction between chronotype and math grades was marginally not significant (*β* = −0.173, 95% CI = −0.343–0.006, *t* = −1.959, *P* = 0.050; Supp. Table 9). The main effect of chronotype does not reach significance (chronotype effect, *F*_1406_ = 3.050, *P* = 0.081, partial *η*2 = 0.026, 90% CI = 0.000–0.027; Supp. Table 8). Importantly, we found a significant interaction between chronotype and school timing (synchrony effect, *F*_1405_ = 7.260, *P* = 0.027, partial *η*2 = 0.016, 90% CI = 0.000–0.040). Consequently, grade retention is significantly affected by the synchrony effect and not by the chronotype effect (Fig. 2).

In order to evaluate which conditions are associated with higher grade retention we calculated the odd ratios from the slopes associated with each combination of predictors (see Methods). For example, the OR associated with MSFsc (without considering the interactions) is the ratio between the grade retention odds of a 1st year student with a specific value of MSFsc (e.g. 07:00) and the odds of another 1st year student with a 1 h earlier MSFsc (e.g. 07:00–1 h = 06:00). That is, if the OR is higher than 1, the student with a 1h-later chronotype would present higher odds of undergo grade retention (e.g. a OR of 1.5 implies 50% higher odds of not reaching 5th year in the expected time).

The inclusion of interactions on this type of models, as in our selected model, means the ORs associated with a variable (e.g. MSFsc) differs according to the values of other variable (e.g. math grades). Consistently, we considered all the interactions included in the selected model (i.e. MSFsc*school timing, math grades*MSFsc, Language grades*School timing) and we calculated the odd ratios (OR) associated to each explanatory variable when the other explanatory variable takes different values (Table 2). For example, the inclusion of the interaction between school timing and chronotype implies that the ORs associated with MSFsc are not the same for students attending to different school timings. Please note that we considered three different values of math grades for MSFsc ORs depending on school timing, because MSFsc also interacts with math grades (Table 2a–c).

**Table 2:** Grade retention depends on the interplay between chronotype, school timing and grades.

	OR	Lower CI	Upper CI
a- MSFsc (math grades 1-point-lower than average)			
Morning*^3^	1.656	1.121	2.447
Afternoon	1.168	0.815	1.676
Evening^1^	0.949	0.728	1.237
b- MSFsc (average math grades)			
Morning^3^	1.393	0.913	2.124
Afternoon	0.983	0.640	1.508
Evening^1^	0.798	0.577	1.105
c- MSFsc (math grades 1-point-higher than average)			
Morning^3^	1.172	0.701	1.958
Afternoon	0.827	0.480	1.425
Evening^1^	0.672	0.429	1.052

	$\frac{1}{{OR}}$	Lower CI	Upper CI
d- Math grades			
1h-earlier MSFsc	1.359	1.859	0.993
Mean MSFsc*	1.616	2.137	1.218
1h-later MSFsc*	2.278	2.717	1.425
e- Language grades			
Morning*^3^	3.106	5.464	1.700
Afternoon*	2.000	3.165	1.267
Evening*^1^	1.553	2.217	1.088

Odd ratios (OR) are $\frac{{{odds}}_{{pred}+1}}{{{odds}}_{{pred}}}$ where var is one of the predictors under study (e.g. MSFsc). In this study ORs represent the relation between the odds of a student of experienced grade retention if his/her MSFsc gets 1h later (e.g. from 05:00 to 06:00 or from 07:00 to 08:00). For grades (i.e. qualifications of math or language), we used $\frac{1}{{OR}}$ ($\frac{1}{{OR}}=\frac{{{odds}}_{{pred}-1}}{{{odds}}_{{pred}}}$) because it facilitates the exposition of the results as they are interpreted as the odds of a student to experience grade retention if her/his grades decrease 1 point. a- MSFsc OR for the three school timings (morning, afternoon, evening) for students with math grades 1-point lower than average (5.15). b- MSFsc OR for the three school timings for students with average math grades (6.15). c- MSFsc OR for the three school timings for students with math grades 1-point higher than average (7.15). d- Math grades $\frac{1}{{OR}}$ for three different chronotypes: average (06:10), 1h-earlier (05:10) and 1h-later than average (07:10) chronotypes. e- Language grades $\frac{1}{{OR}}$ for each school timing. Each OR ($\frac{1}{{OR}}$) (significantly different to 1 is indicated by an asterisk (*). Additionally, a superscript number indicates if it is different from other/s ORs ($\frac{1}{{ORs}}$): 1-compared with morning; 3-compared with evening. MSFsc = corrected midpoint of sleep on free days.

*MSFsc* corrected midpoint of sleep on free days, *OR* odd ratio, *CI* confidence Interval.

Interestingly, the odds that a student with a 1h-later chronotype experience grade retention are higher in the morning than in the evening and the extent of this effect is higher when math grades are lower (Tables 2a–c). Specifically, when comparing two students with math grades of 5.15 (1-point-lower than average) that differ in their chronotypes by 1 h (e.g. 05:10 vs. 06:10), the student with later chronotype shows 1.656 higher odds (i.e. 66%) of experiencing grade retention when attending the morning school timing (Table 2a). MSFsc OR are not significantly different from one in any school timing for students with average or 1-point-higher math grades (Table 2b, c, respectively). Regarding math grades, a student with 1 point-lower grade and an average chronotype (i.e. 06:10) would have 1.616 significant higher odds of repeating a grade (Table 2d). This effect would not be significant for a 1h-earlier chronotype (i.e. 05:10), but it would be stronger for a 1h-later chronotype (i.e. 07:10), who would have 2.278 higher odds of grade retention for 1-point-lower math grades (Table 2d). Finally, lower language grades increase the odds of grade retention in all school timings but this effect is stronger for morning than for evening-attending students. In particular, in morning school timing a 1-point-lower language grade is associated with 3.106 higher odds of grade retention while in the evening the odds are 1.553 higher (Table 2e).

## Discussion

Here we studied whether and how chronotype and/or its alignment with school timing affect academic success, including not only academic performance (i.e. grades) but also grade retention. Even though a chronotype effect on academic success could not be ruled out, our results show that both academic performance and the odds of experiencing grade retention are influenced by the interaction between chronotype and school timing (i.e. synchrony effect). Particularly, morning-attending students with later chronotypes tend to present lower academic performance and higher odds of repeating a grade than their peers with earlier chronotypes. Contrastingly, the association with chronotype in both afternoon- and evening-attending students is weaker for academic performance and absent for grade retention.

We found a main effect of chronotype on academic performance, with late chronotypes exhibiting lower grades. However, the magnitude of this effect depends on age, school subject and, importantly, on school timing, which is evident considering the significant double and triple interactions between these factors, even though the quadruple interaction was not significant. These results indicate that both chronotype and synchrony effects impact on academic performance but in different ways. First, and consistent with previous reports^48,49,56^, math performance seems to be more affected by chronotype and its alignment with school timing than language (i.e. native language: Spanish) performance. This difference could be explained by the fact that math relies more on fluid intelligence while language, in crystallized intelligence, and that the former has been reported to be more affected by both the chronotype and the synchrony effects^46,47^ than the latter. However, language grades are consistently higher than math grades and present lower variability in our data, especially in 5th year, and thus a ceiling effect could be masking the chronotype and synchrony effects on this school subject. Second, both chronotype and synchrony effects are more evident when adolescents are older. In 1st year, the afternoon is the only school timing where chronotype is not associated with academic performance; that is, earlier chronotypes do not have an advantage over later chronotypes. In 5th year, despite the fact that chronotype is associated with academic performance in all school timings, the magnitude of the effect for evening and afternoon school timings is similar. In addition, when comparing mean grades between school timings (i.e. without considering each student’s chronotype) we observed similar results: in 1st year, afternoon-attending students presented the highest math grades, but in 5th year evening-attending students outperformed their peers. Together, these results suggest that during their 1st year of school, students are better aligned to the afternoon school timing than to the evening school timing, but this difference disappears or even reverses during their last year. This might be because chronotypes become later during adolescence^7,8,56,57^ but school start times do not change. As a result, the misalignment between students’ internal timing and earlier school schedules increases with age. Finally, the magnitude of the association between chronotype and academic performance differs between school timings, which evidences the existence of a synchrony effect. Importantly, previous works have suggested the presence of this effect^38–40,52,62^ but their study conditions were insufficient to determine whether the synchrony and/or the chronotype effect explain their academic performance results. The experimental setup presented here and in our previous cross-sectional study^56^ (i.e. including three different school timings to which students were randomly assigned) allows us to further address this issue (see Supp. Discussion for the comparison between both studies). In our results, the existence of the synchrony effect is particularly clear in math performance during 5th year, where the association between later chronotypes and lower academic performance is higher in the morning than in both afternoon and evening school timings. Nevertheless, the chronotype effect is also present: earlier types still perform better than later types in the evening. Hence, the synchrony effect appears to be not strong enough to revert or cancel the chronotype effect. Therefore, both the chronotype effect and the synchrony effect modulate the interplay between chronotype, school timing and academic performance. Importantly, the synchrony effect could be acting at different levels and thus, not showing all its strength in the modulation of the relation between chronotype and grades. For example, we observed indirect evidence of a synchrony effect affecting academic performance when comparing grades between school timings, as humans present late chronotypes during adolescence it was expected that afternoon and evening attending students presented better academic performance than their morning attending peers. Importantly, the synchrony effect could be affecting academic success through other mechanisms or outcomes, such as influencing motivation^50,63–65^ or, as studied here, impacting grade retention (i.e. students’ difficulty to complete their studies in a timely manner).

Regarding grade retention, we observed that students with consistently lower grades in math or/and language in their 1st year present higher odds of repeating at least a grade during secondary school. The latter was expected considering that the criterion for deciding whether a student should repeat a grade is mainly based on their academic performance. Interestingly, even though the interaction between math performance and chronotype on grade retention was not significant, it was relevant: students with 1-point lower math grades did show higher odds to grade retention when their chronotypes are equal or later than the average, but not when their chronotypes were 1 h earlier. This shows that chronotype effect is affecting academic success in other ways besides influencing academic performance. Moreover, the interaction of chronotype with school timing clearly shows that the synchrony effect can affect grade retention. The odds of grade retention associated with chronotype are different between morning and evening school timings, even considering different math grades. A student with later chronotype attending school in the morning shows significantly higher odds of experiencing grade retention than an evening-attending later chronotype. Although not always significant, the ORs of experiencing grade retention related to MSFsc (i.e. MSFsc OR) show different tendencies depending on school timing according to the synchrony effect: in the morning, later students show higher odds of repeating a grade while in the evening, earlier students show higher odds of grade retention. This suggests that synchrony effect is influencing grade retention.

Little is known about how adolescents’ unhealthy sleep habits affect grade retention^58,59^. The collision between early school start times and adolescents’ late chronotypes could be the cause of the mentioned unhealthy sleep habits, however, our results also add evidence about a link between chronotype (alone and interacting with school timing) and grade retention. This is particularly important because most studies use academic performance as the only proxy of academic success^35,49,51,52,56^ and this outcome is probably also affected by grade retention. For example, in this longitudinal study we only consider those students for which we had data from both 1st and 5th year when assessing academic performance. However, those students experiencing grade retention, probably because of their low grades, were unable to timely reach their 5th year and, thus, they were not included in the academic performance analyses. This means that the literature, in general, is probably underestimating the effect of students’ internal timing on their school outcomes. Consistently, the lack of a strong synchrony effect in academic performance could be partially explained by grade retention results: morning-attending students are more prone than evening-attending students to repeat a grade if they show lower language grades in 1st year. That is, less morning-attending students with lower grades would reach 5th year. Then, only including academic performance could not be enough to capture chronotype and synchrony effects on academic success, as morning-attending students experience higher grade retention. However, the latter is not necessarily the case for evening-attending students, whose grades and chronotype are weaker predictors of grade retention, and thus, academic performance could be a more useful outcome to capture chronotype and synchrony effects on academic success. Altogether, our results show that the mechanisms and pathways by which both chronotype and synchrony effects influence academic success are varied and more complex than previously reported.

This study has several limitations. First, academic performance was measured as grades that were assigned by teachers who are not blind to students’ identity and who differ between courses. Relatedly, the teachers’ chronotype was unknown and it could affect their performance if it is not aligned with the school timing where they work. However, these biases are highly improbable to affect our results given the number of courses included, the large number of teachers, and the fact that several of them work in more than one school timing. Second, chronotype was assessed using self-reported questionnaires and future works could benefit from the usage of more objective methods to estimate chronotype, such as actigraphy. Third, results are based on correlations, which do not allow us to establish causality relationships. Nonetheless, as students are randomly assigned to their school timing at the start of secondary school, we can assume that basal distributions of chronotype and academic performance were similar between school timings. Finally, we cannot completely differentiate between grade retention and a change of school, or even school dropout, as we only know which 1st year students in 2015 do not reach 5th year in 2019. However, as the school is very prestigious and just a small fraction (~25%) of the adolescents who apply are admitted, it is highly unusual that students decide to dropout or change schools once they have managed to enter this school.

Importantly, this work also has specific and important strengths. First, we included three different school timings that cover a wide range of the day and allow us to test the chronotype versus the synchrony effect. Second, we included two age points, one at the start and the other at the end of secondary school, allowing us to study how the interplay between chronotype and time of day affects academic achievement at different and distant ages during adolescence. Third, this is a longitudinal study, which allow us to control for within-subject variability and, thus, obtain stronger conclusions about the contribution of age in our observations. Fourth, thanks to the longitudinal character of the study, we not only assessed academic performance but also grade retention which is a very relevant outcome related to academic success. Finally, the random assignment of students to one of the three different school timings at the start of secondary school lets us assume an unbiased distribution of chronotype and academic success between school timings.

This study has several practical implications. First, and consistent with previous reports^35,49,51,52,56^, we observed a disadvantage of later chronotypes over earlier chronotypes regarding academic success, especially when attending the morning school timing. These results suggest that delaying start times could be a possible policy to mitigate this disadvantage, as some previous reports showed^35,36,66^, especially for those students with later chronotypes at the beginning of secondary school. Second, we observed a stronger disadvantage for math performance in older students. Thus, if the school timing delay policy should be applied only to some students, older students probably will obtain higher benefits. Also, based on the results presented here, another policy could be assessing students’ chronotype before starting their first year of secondary school and assign those with later chronotypes to later school timings, even considering that the change in chronotype throughout secondary school also depends on baseline chronotype^57^. Another practical implication of our results is the possibility of reorganizing the order of school subjects, letting math for the last hours of the school day, particularly during morning school timing. Finally, grade retention is a controversial practice, because it has been reported to be associated with higher school dropout^67–69^, lower academic performance^60,70^, lower earnings in post-high school labor market^67^ and deepens existing inequalities (e.g. lower socioeconomic status correlates with higher grade retention)^71,72^. Here, we add novel evidence indicating that grade retention is not equally fair when students’ chronotype is considered, which strongly supports the idea of rethinking grade retention as an educative practice^73,74^. Although we propose some important practical implications of our findings, we want to emphasize the importance of translational studies to test possible applications, and the necessity of more evidence, especially local evidence, to strengthen our conclusions and to design educational public policies. Altogether, it is important to highlight that early school start times not only disadvantage students with later chronotypes but also affect most adolescents’ sleep habits and well-being^8,35,56,57,75,76^. Consistently, we think it is time that the chronobiology scientific community, policy makers and educators start interacting to think and evaluate possible implementations to address these issues, to improve adolescents’ education and health, and to better prepare teenagers for the future.

## Methods

### Ethical approval

The study and all the methods included were conducted following the ethical recommendations for human chronobiological research^77^ and Argentinian national regulations^78^. In particular, Argentinian laws consider that adolescents of at least 13 years of age can decide over their own body as long as the activity or procedure is not invasive and does not pose a serious risk to their life or physical integrity, as is the case of this study. In addition, due to Argentinian regulations, parents accept that the school institution has the authority to decide the curricular activities in which the students will take part, and the school authorities approved this study as a curricular activity. Consistently, a written informed consent was obtained from the head of the school, while parents’ written consent was not required. Importantly, students were clearly informed about the voluntary nature of their participation, they knew they could leave the activity at any time without any consequences, and that completing the questionnaire implied they accepted to participate in the study. Students provided oral, but not written, and active informed consent to participate. The protocol for this study was approved by the institutional Ethical Committee of the Universidad Nacional de Quilmes (Verdict #4/2017).

### Participants

This study was performed in two different moments (June 2015 and July 2019) at a local secondary school in the City of Buenos Aires, Argentina (34° 60′ S, 58° 38′ W). In Buenos Aires, the school year starts in March and ends in December and, thus, the data were collected after three/four months of classes on the corresponding academic year. All 1st year (i.e. 2015) or 5th year (i.e. 2019) students who attended school the day of data collection were invited to participate in the study. Students are distributed in different classrooms: there are five classrooms or groups of students for each school year in each school timing, that is, in this study we consider 30 different classrooms (15 for 1st year and 15 for 5th year). The attendance percentage was higher than 75% on each school timing and year (2015: morning, 97.50%; afternoon, 90.24%; evening, 87.01%. 2019: morning, 75.35%; afternoon, 79.11%; evening, 91.23%) and no student refused to participate.

In the analysis including chronotype and academic performance, from the 436 and 352 students who completed the questionnaire in their 1st and 5th year, respectively, 259 students were included. Only those students who participated in the study in both years, who maintained their original school timing and with complete data (i.e. MSFsc, school timing, age and school grades) in both years were included. The resulting sample of students was balanced on gender (50.97% females) and age-homogeneous (1st year: *M* = 13.49 y.o., S*d* = 0.33; 5th year: *M* = 17.58 y.o., S*d* = 0.33). Mean chronotype, SJL, sleep duration and sleep timings on weekdays and free days are presented in Supp. Table 10.

In the analysis regarding chronotype and grade retention, the 407 students that presented complete data in their 1st year were included. The resulting sample of students was balanced on gender (49.88% females). Here, we assume that the main reason for students starting their 1st year in 2015 and not reaching 5th year in 2019 is that they experienced grade retention and not other reasons such as a change of school, school dropout or because parents moved to another city or country. This assumption is based on: (1) it is very difficult to be accepted in this school: students should do a 1 year-course and they should obtain good qualifications to be one of the ~500 best students that will be accepted, among the ~2000 students that participate in the course; (2) the school has an excellent academic level and it is one of the five secondary schools that are administered by the prestigious Universidad de Buenos Aires (UBA, Buenos Aires University). Therefore, it is very unlikely that students will decide to change to another school if it is not because they are struggling academically.

### Procedure

One important aspect of our experimental setup is that in this particular school, there are three different school timings (morning, 07:45–12:05; afternoon, 12:40–17:00; evening, 17:20–21:40). The other important aspect is the assignation to one of the school timings is decided by a lottery system at the beginning of the secondary school and they maintain their originally assigned school timing throughout their secondary school, as described in depth in our previous study^56^.

Students filled a questionnaire including demographic information (date of birth and self-defined gender) and the Spanish version of the MCTQ^17^ from which we obtain a local time point (MSFsc) as a proxy of chronotype. Earlier times or lower values of MSFsc indicate earlier chronotypes, and later times (i.e. higher values of MSFsc) indicate later chronotypes. Data collection was performed during students’ typical school hours (morning, afternoon and evening school timings, respectively). Grades and lists of students were obtained at the end of the academic year. Data collection and analysis were not performed blind to the conditions of the experiments. The same procedure was applied in both June 2015 (during students’ first school year) and July 2019 (during their last school year).

### Measurements

For each student in each school year, we obtained: the sleep-corrected midpoint of sleep time on free days (MSFsc)^17^, grades for each school subject ranging from 1 (lowest/worst) to 10 (highest/best) and information on whether they reached 5th year or not.

All grades throughout the whole academic year were obtained from the school registers. Each student has four different grades or qualifications for each school subject and year: two general grades and two integrative grades (one of each type for each half of the academic year). General grades are decided by the teacher considering student performance during classes and in small tests. Integrative grades are derived from comprehensive exams. When we refer to ‘type of grade’ throughout the manuscript, we are referring to these two categories. To pass a school subject, two conditions must be fulfilled: (1) a minimum grade of four on each integrative exam and (2) the average of the four grades must be ≥6.5.

Here, we only consider school subjects imparted during the corresponding school timing hours (a few school subjects, such as physical education, are imparted outside the expected hours for the corresponding school timing^56^). Most school subjects vary between 1st and 5th year. In particular, only math and language (Spanish, which is their native language) are present in both years. Thus, we aggregate subjects in three categories: math, language and other subjects. When controlling for the effect of academic performance in grade retention only math and language were included.

Missing values occurred when a variable could not be calculated because of incomplete information (e.g. when a student did not complete all of the MCTQ questions). The data from a student was only included if the information was complete.

### Statistical analysis

All statistical analyses were performed using the R system for statistical computing (v.4.0.2; R Core Team, 2020).

We ran linear mixed-effect models to determine whether chronotype (as numeric) interacting with school timing (as factor: morning, afternoon or evening), age (as factor: 1st or 5th year) and school subject (as factor: math, language or other subjects) modulates academic performance (as numeric: from 1 to 10). Grades of the whole academic year were included in the model. We controlled for gender including it as a fixed factor. Student ID nested within classroom (as factor: 1–30) and type of grade (as factor: general or integrative) were included as random factors. That is, in R software: lmer(Grades ~ MSFsc × School subject × School timing × School year + Gender + (1|Student’s ID) + (1 | Classroom/Student’s ID) + (1 | type of grade)). *P*-values were computed using lmerTest package^79^.

In order to find the best model explaining the relation between school timing, grades and chronotype we ran a set of logistic regression models and chose the one with the lowest Akaike. Grade retention was included as a binary categorical variable reflecting whether each student reached the 5th year on the expected time (grade retention = 0) or not (grade retention = 1). The null/base model includes math and language grades (as grades are good predictors of grade retention), chronotype (as numeric), and the interaction between chronotype and school timing (as factor), as we expected a synchrony effect between chronotype and school timing. Grades are included as a numeric variable (1–10) and they are the average of the four grades for each subject (math and language). The most parsimonious model includes math and language grades and chronotype as main factors and the interactions between chronotype and school timing, chronotype and math grades and school timing and language grades. That is, in R software: glm(Grade retention ~ MSFsc + Math grades + Language grades + Language grades:School timing + Math grades:MSFsc + School timing:MSFsc, family = “binomial” (link = ‘logit’). We calculated the odd ratios for each combination of predictors from the slopes of this model (OR = e^slope^). In the case of school grades, we used $\frac{1}{{OR}}$ as they are interpreted as the odds of a student to experience grade retention if her/his grades decrease 1 point, which simplifies the results interpretation.

### Reporting summary

Further information on research design is available in the Nature Research Reporting Summary linked to this article.

## Supplementary information

Supplementary Information

Reporting Summary

## Data Availability

The data that support the findings of this study are available from the corresponding author on request.

## Code availability

The code that supports the findings of this study are available from the corresponding author on request.
