# Supplementary material for: A better alignment between chronotype and school timing is associated with lower grade retention in adolescents
Source: NPJ Sci Learn. 2023 Jun 21;8:21. doi: 10.1038/s41539-023-00171-0 (PMC10284813; doi:10.1038/s41539-023-00171-0)
Supplement: Supplementary file 1 — Supplementary Information [file 41539_2023_171_MOESM1_ESM.pdf]

Supplementary information

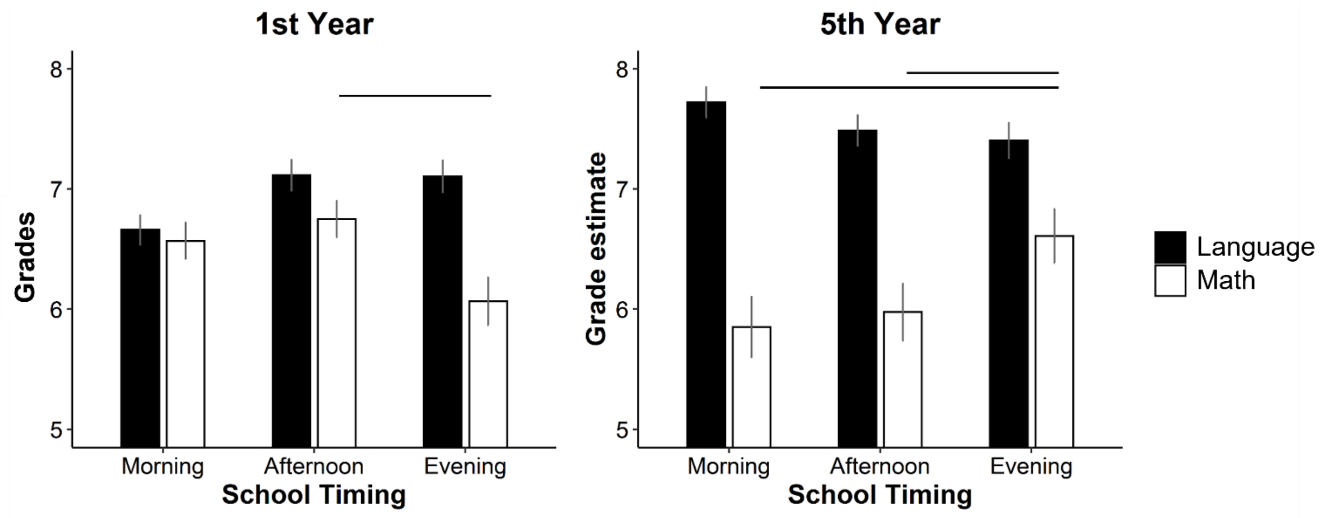

Supplementary Figure 1. Grades depend on school timing, age and school subject. Grade means (± SEM) for 1st and 5th year students attending to morning, afternoon or evening school timing. Age was entered as a factor (for 1st or 5th year). Only comparisons between school timings within each school year and school subject were performed. The lines on top of the bars indicate the significant pairwise comparisons between school timings. n = 259.

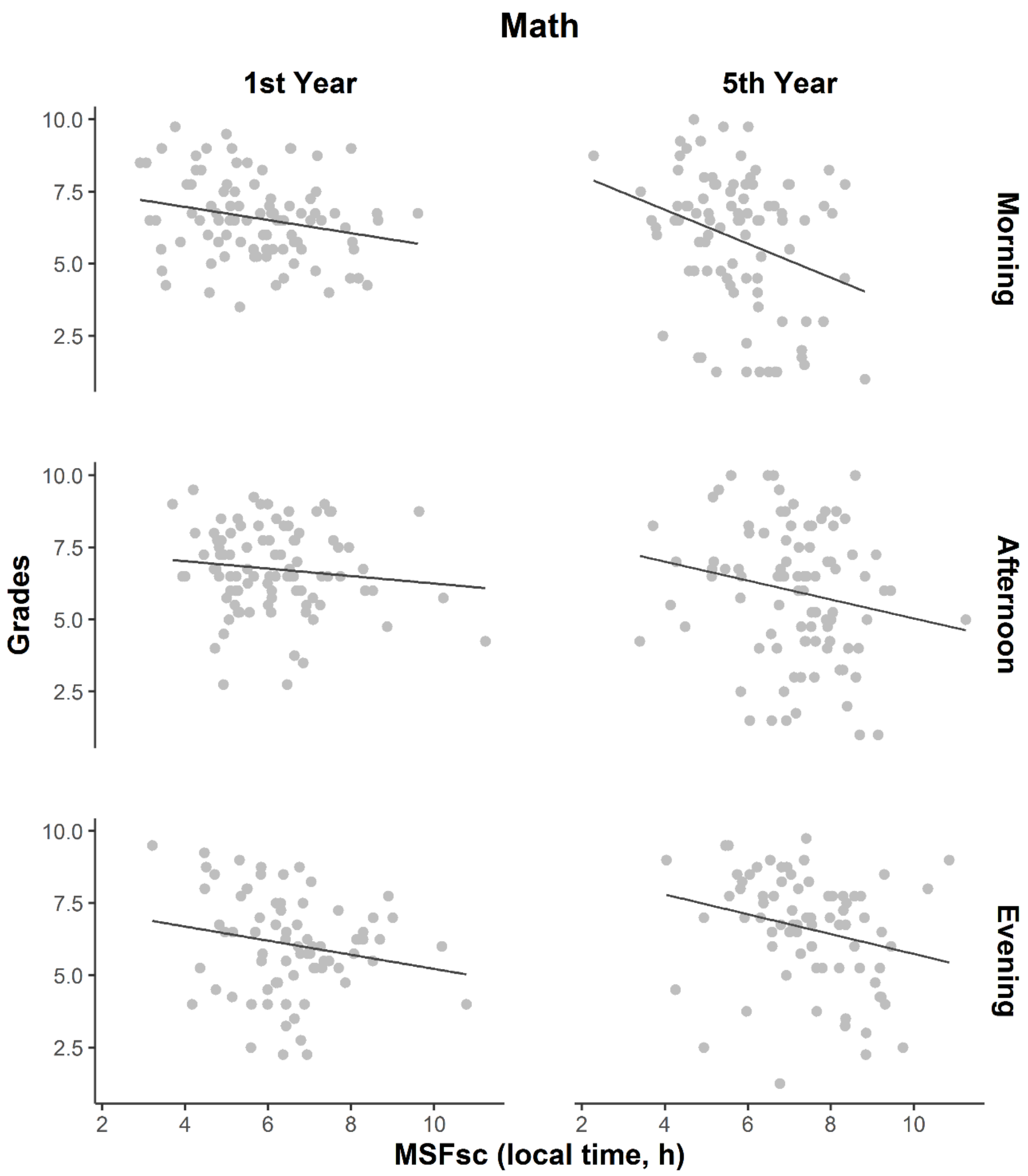

Supplementary Figure 2. Relationship between math grades and chronotype. Each dot represents the intersection between MSFsc and the average of each student's grades. The left panels show data from 1st year and the right panels, from 5th year students. Morning, Afternoon and Evening school timing data is plotted in first to third rows, respectively. X-axis: Midpoint of Sleep on Free days, sleep corrected (MSFsc); all values are represented in hours (local time, h). Y-axis: Mean value of the four grades given in Math to a student during the whole school year. The corresponding regression line is drawn in each panel. n = 259.

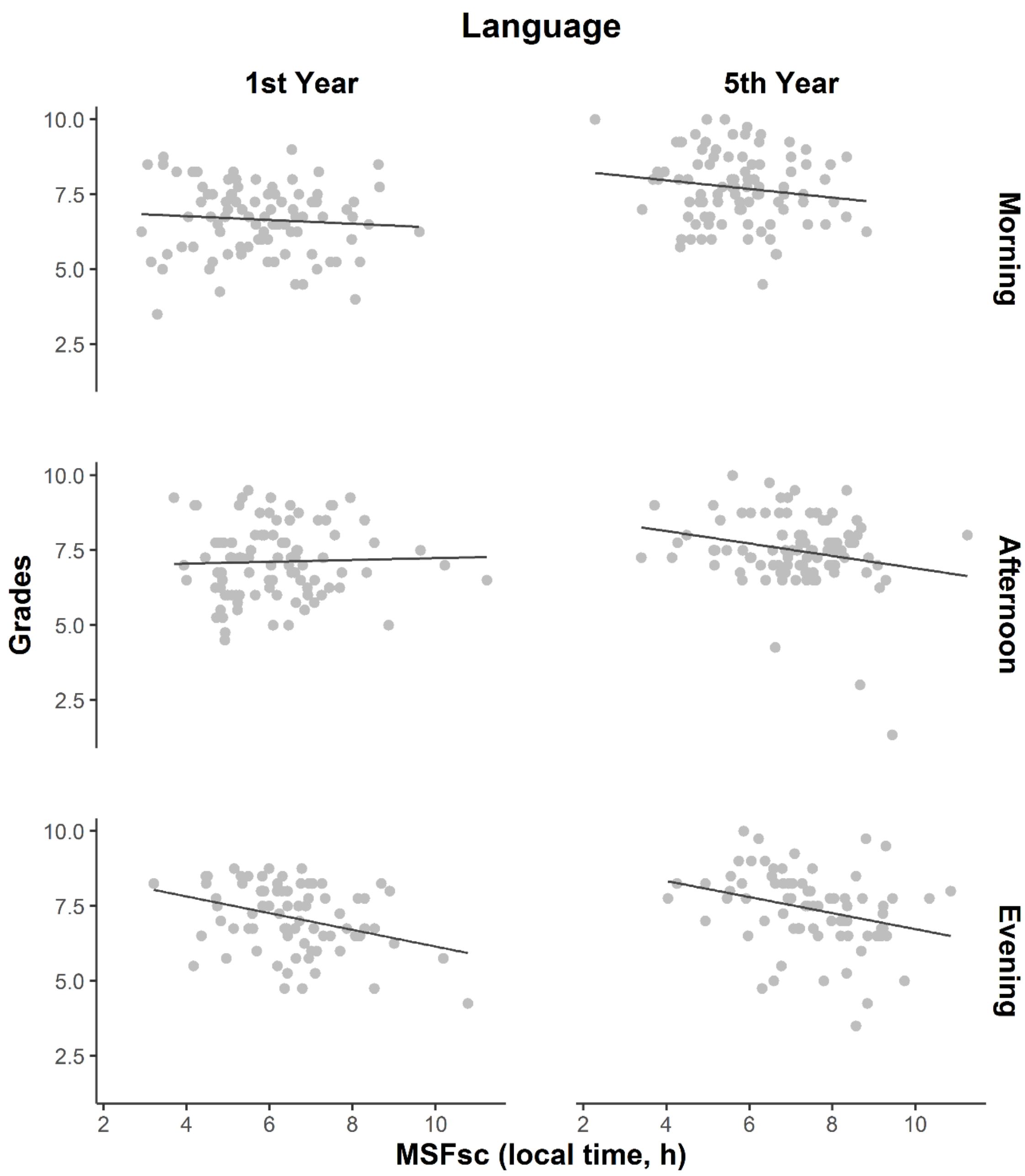

Supplementary Figure 3. Relationship between Language grades and chronotype. Each dot represents the intersection for one student. Students in 1st year in the left column, students in the 5th year in the right column. Morning, Afternoon and Evening school timings in first to third rows, respectively. X-axis: Midpoint of Sleep on Free days, sleep corrected (MSFsc); all values are represented in hours (local time, h). Y-axis: Mean value of the four grades given in Language to a student during the whole school year. The corresponding regression line is drawn in each panel. n = 259.

Supplementary Table 1. Demographic and grades information. Grades means and their standard deviations were calculated for each school year, school timing and gender. Age was evaluated as a factor (for 1st or 5th year). n = 259.

**Age group**	**School timing**	**Gender**	**Grades (M (SD))**	
			**Language**	**Math**
1st Year	Morning	Female	6.898 (1.201)	6.740 (1.292)
		Male	6.381 (1.231)	6.369 (1.552)
	Afternoon	Female	7.378 (1.379)	7.000 (1.592)
		Male	6.885 (1.018)	6.535 (1.325)
	Evening	Female	7.400 (0.935)	6.219 (1.690)
		Male	6.771 (1.246)	5.892 (1.760)
5th Year	Morning	Female	7.910 (1.377)	6.180 (2.284)
		Male	7.488 (0.868)	5.451 (2.450)
	Afternoon	Female	7.881 (0.950)	6.295 (2.472)
		Male	7.134 (1.323)	5.689 (2.113)
	Evening	Female	7.641 (1.225)	6.673 (2.020)
		Male	7.146 (1.398)	6.542 (1.866)

Supplementary Table 2. ANOVA table for the model explaining academic performance as a function of school timing, age and school subject. n = 259. Sum Sq, sum of squares.

	**Sum Sq**	**d.f**	**F**	**P**	**Partial η2**	**90% CI**
age	2.339	1	0.908	0.357	0.062	[0.000-0.331]
School timing	1.156	2	0.225	0.800	0.009	[0.000-0.062]
School subject	2590.532	2	503.070	<0.0001	0.056	[0.051-0.062]
School timing:Age	13.122	2	2.548	0.145	0.271	[0.000-0.518]
School subject:Age	214.917	2	41.736	<0.0001	0.005	[0.003-0.007]
School subject:School timing	12.080	4	1.173	0.320	0.000	[0.000-0.001]
School subject:School timing:Age	185.045	4	17.967	<0.0001	0.004	[0.003-0.006]

Supplementary Table 3. Pairwise comparisons of grades between school timings. Pair-wise [(1) – (2)] comparisons of grades were tested for those pairs of different school timings, while keeping age and school subject constant. n = 259.

Age group	School subject	School Timing (1) (2)		t-value	P	Cohen's d	95% CI
1st Year	Others	Morning	Afternoon	-0.837	0.680	-0.095	[-0.317 to 0.127]
1st Year	Others	Morning	Evening	0.295	0.953	0.034	[-0.194 to 0.263]
1st Year	Others	Afternoon	Evening	1.110	0.508	0.129	[-0.099 to 0.357]
1st Year	Language	Morning	Afternoon	-2.177	0.075	-0.287	[-0.545 to -0.029]
1st Year	Language	Morning	Evening	-2.183	0.074	-0.298	[-0.595 to -0.030]
1st Year	Language	Afternoon	Evening	-0.083	0.996	-0.011	[-0.278 to 0.256]
1st Year	Math	Morning	Afternoon	-0.888	0.648	-0.117	[-0.375 to 0.141]
1st Year	Math	Morning	Evening	2.155	0.079	0.294	[0.027 to 0.561]
1st Year	Math	Afternoon	Evening	3.018	0.007	0.411	[0.144 to 0.677]
5th Year	Others	Morning	Afternoon	0.536	0.854	0.060	[-0.160 to 0.281]
5th Year	Others	Morning	Evening	0.068	0.997	0.008	[-0.219 to 0.235]
5th Year	Others	Afternoon	Evening	-0.454	0.893	-0.053	[-0.279 to 0.174]
5th Year	Language	Morning	Afternoon	1.080	0.526	0.142	[-0.116 to 0.400]
5th Year	Language	Morning	Evening	1.478	0.301	0.202	[-0.066 to 0.469]
5th Year	Language	Afternoon	Evening	0.436	0.900	0.059	[-0.207 to 0.326]
5th Year	Math	Morning	Afternoon	-0.550	0.846	-0.072	[-0.330 to 0.186]
5th Year	Math	Morning	Evening	-3.432	0.002	-0.468	[-0.736 to -0.201]
5th Year	Math	Afternoon	Evening	-2.908	0.010	-0.396	[-0.663 to -0.129]

Supplementary Table 4. ANOVA table for the model explaining academic performance as a function of MSFsc, school subject, school timing, age and gender. Significant interactions imply changes in the slope and/or the intercept of the regression lines between MSFsc and grades. Specifically, interactions between MSFsc and other predictor(s) are associated with changes in the slope. Interactions among other predictors are associated with changes in the intercept. Retrospective power for the four-way interaction in this model was 90% (approximated using bootstrapping); n = 259. Sum Sq, sum of squares. To obtain a more natural interpretation of the model's estimates, MSFsc was included relative to its global mean (M= 06:27).

	Sum Sq	d.f	F	P	Partial η2	90% CI
MSFsc	64.796	1	25.264	<0.0001	0.041	[0.019-0.070]
School subject	2203.130	2	429.501	<0.0001	0.048	[0.043-0.054]
School timing	11.812	2	2.303	0.110	0.082	[0.000-0.205]
Age	5.810	1	2.265	0.149	0.110	[0.000-0.355]
Gender	54.311	1	21.176	<0.0001	0.074	[0.031-0.129]
MSFsc:School subject	102.361	2	19.955	<0.0001	0.002	[0.001-0.004]
MSFsc:School timing	4.578	2	0.893	0.410	0.003	[0.000-0.012]
School subject:School timing	75.500	4	7.359	<0.0001	0.002	[0.001-0.003]
MSFsc:Age	10.958	1	4.273	0.039	0.009	[0.000-0.029]
School subject:Age	171.307	2	33.396	<0.0001	0.004	[0.002-0.006]
School timing:Age	16.663	2	3.248	0.062	0.262	[0.000-0.485]
MSFsc:School subject:School timing	56.832	4	5.540	<0.001	0.001	[0.000-0.002]
MSFsc:School subject:Age	33.871	2	6.603	0.001	0.001	[0.000-0.002]
MSFsc:School timing:Age	3.320	2	0.647	0.524	0.003	[0.000-0.013]
School subject:School timing:Age	187.203	4	18.248	<0.0001	0.004	[0.003-0.006]
MSFsc:School subject:School timing:Age	17.213	4	1.678	0.152	0.00	[0.000-0.001]

Supplementary Table 5. Summary of the model explaining academic performance as a function of MSFs, school subject, school timing, age and gender. Reference levels: Morning, Other subjects, young and Female. n = 259.

		Beta	95% CI	t-value
1	Intercept	7.655	[7.398 to 7.916]	54.00
2	MSFsc	0.006	[-0.091 to 0.104]	0.12
3	Language	-0.843	[-1.041 to -0.646]	-8.36
4	Math	-1.048	[-1.245 to -0.850]	-10.39
5	Afternoon	0.146	[-0.172 to 0.464]	0.83
6	Evening	-0.051	[-0.380 to 0.272]	-0.28
7	5th Year	0.052	[-0.223 to 0.326]	0.34
8	Male	-0.403	[-0.575 to -0.232]	-4.60
9	MSFsc:Language	-0.042	[-0.166 to 0.082]	-0.66
10	MSFsc:Math	-0.206	[-0.330 to -0.082]	-3.26
11	MSFsc:Afternoon	-0.100	[-0.242 to 0.041]	-1.37
12	MSFsc:Evening	-0.111	[-0.271 to 0.034]	-1.43
13	Language:Afternoon	0.394	[0.126 to 0.663]	2.88
14	Math:Afternoon	0.183	[-0.085 to 0.451]	1.34
15	Language:Evening	0.569	[0.291 to 0.848]	4.01
16	Math:Evening	-0.270	[-0.548 to 0.009]	-1.90
17	MSFsc:5th Year	-0.040	[-0.178 to 0.096]	-0.57
18	Language:5th Year	0.967	[0.683 to 1.252]	6.66
19	Math:5th Year	-1.017	[-1.301 to -0.732]	-7.00
20	Afternoon:5th Year	-0.156	[-0.540 to 0.229]	-0.72
21	Evening:5th Year	0.187	[-0.209 to 0.587]	0.84
22	MSFsc:Language:Afternoon	0.216	[0.036 to 0.395]	2.35
23	MSFsc:Math:Afternoon	0.221	[0.041 to 0.400]	2.41
24	MSFsc:Language:Evening	-0.038	[-0.227 to 0.151]	-0.40
25	MSFsc:Math:Evening	0.160	[-0.029 to 0.349]	1.66
26	MSFsc:Language:5th Year	-0.016	[-0.207 to 0.176]	-0.16
27	MSFsc:Math:5th Year	-0.296	[-0.487 to -0.104]	-3.03
28	MSFsc:Afternoon:5th Year	0.090	[-0.102 to 0.286]	0.91
29	MSFsc:Evening:5th Year	0.035	[-0.156 to 0.238]	0.35
30	Language:Afternoon:5th Year	-0.436	[-0.827 to -0.046]	-2.19
31	Math:Afternoon:5th Year	0.534	[0.143 to 0.924]	2.68
32	Language:Evening:5th Year	-0.785	[-1.206 to -0.364]	-3.65
33	Math:Evening:5th Year	1.526	[1.105 to 1.947]	7.10
34	MSFsc:Language:Afternoon:5th Year	-0.228	[-0.495 to 0.038]	-1.68
35	MSFsc:Math:Afternoon:5th Year	0.074	[-0.192 to 0.341]	0.55
36	MSFsc:Language:Evening:5th Year	0.043	[-0.234 to 0.320]	0.30
37	MSFsc:Math:Evening:5th Year	0.213	[-0.064 to 0.490]	1.51

Supplementary Table 6. Slope pair-wise comparisons (MSFsc model). Pair-wise [(1) – (2)] comparisons of slopes were tested for those pairs corresponding to different levels of one factor, while keeping the others constant (e.g. the slope of the association between MSFsc and Math grades on younger students attending different school schedules). t-values are considered significant when their absolute values are higher than 2. n = 259.

Age group	School subject	School Timing (1)	(2)	t-value	Cohen's d	95% CI
1st Year	Others	Morning	Afternoon	1.369	0.062	[-0.027 to 0.151]
1st Year	Others	Morning	Evening	1.427	0.070	[-0.026 to 0.165]
1st Year	Others	Afternoon	Evening	0.146	0.007	[-0.091 to 0.105]
1st Year	Language	Morning	Afternoon	-1.095	-0.072	[-0.202 to 0.057]
1st Year	Language	Morning	Evening	1.326	0.093	[-0.045 to 0.231]
1st Year	Language	Afternoon	Evening	2.302	0.166	[0.025 to 0.307]
1st Year	Math	Morning	Afternoon	-1.142	-0.076	[-0.205 to 0.054]
1st Year	Math	Morning	Evening	-0.434	-0.031	[-0.169 to 0.107]
1st Year	Math	Afternoon	Evening	0.625	0.045	[-0.092 to 0.186]
5th Year	Others	Morning	Afternoon	0.120	0.006	[-0.092 to 0.103]
5th Year	Others	Morning	Evening	0.911	0.047	[-0.055 to 0.149]
5th Year	Others	Afternoon	Evening	0.851	0.041	[-0.054 to 0.137]
5th Year	Language	Morning	Afternoon	0.188	0.014	[-0.131 to 0.158]
5th Year	Language	Morning	Evening	0.583	0.044	[-0.105 to 0.194]
5th Year	Language	Afternoon	Evening	0.424	0.031	[-0.111 to 0.172]
5th Year	Math	Morning	Afternoon	-2.421	-0.178	[-0.323 to -0.034]
5th Year	Math	Morning	Evening	-2.436	-0.186	[-0.335 to -0.036]
5th Year	Math	Afternoon	Evening	-0.102	-0.007	[-0.149 to 0.134]

School timing	School subject	Age group (1)	(2)			
Morning	Language	1st Year	5th Year	0.508	0.035	[-0.099 to 0.168]
Afternoon	Language	1st Year	5th Year	1.808	0.121	[-0.010 to 0.252]
Evening	Language	1st Year	5th Year	-0.201	-0.014	[-0.153 to 0.125]
Morning	Math	1st Year	5th Year	3.070	0.209	[0.076 to 0.343]
Afternoon	Math	1st Year	5th Year	1.597	0.107	[-0.024 to 0.238]
Evening	Math	1st Year	5th Year	0.767	0.054	[-0.084 to 0.193]

Age group	School timing	School subject (1)	(2)			
1st Year	Morning	Math	Language	1.993	0.103	[0.002 to 0.204]
1st Year	Afternoon	Math	Language	1.839	0.100	[-0.007 to 0.206]
1st Year	Evening	Math	Language	-0.356	-0.021	[-0.138 to 0.096]
5th Year	Morning	Math	Language	4.518	0.277	[0.157 to 0.398]
5th Year	Afternoon	Math	Language	1.524	0.085	[-0.024 to 0.195]
5th Year	Evening	Math	Language	0.803	0.047	[-0.068 to 0.163]

Supplementary Table 7. Model comparisons for predictors and their interactions on grade retention. The initial model includes chronotype and its interaction with school timing and math and language grades. First, we added school timing to the model but the AICc does not decrease. Thus, we continued with the initial model and successively added some relevant interactions and if the AICc decreases, we maintain the interaction in the model. Specifically we added the following interactions: a) school subjects grades and school timing, as it also could imply and indirect evidence of the synchrony effect (models 3 and 4); b) school subject grades and MSFsc because, as previously stated, grades can be modulated by chronotype and the modulation can vary with the school subject (models 5 and 6); c) math grades and language grades, as it is possible that presenting lower grades in both school subjects have a greater impact on grade retention than exhibiting lower grades in only one of them (model 7); d) school subject grades, chronotype and school timing as the synchrony effect could be modulated by school subject (models 8 and 9);e) and the quadruple interaction (model 10). The most parsimonious model is the Model 5. MSFsc: Mid-sleep on free days corrected for sleep. Math: Mean grades for math. Language: Mean grades for language. K: degrees of freedom. AICc: Akaike information Criterion corrected. Delta AICc: AICc difference between a model and the best one. AICcWt: AICc weight, which indicates the relative weight of a model relative to the best one. ER: evidence ratio, ER= AICcWt(best)/AICcWt(i). LER: logarithm of evidence ratio, LER= log10 (ER). n=407.

	Explanatory variables	K	AICc	dAICc	Weight	ER	LER
1	Math + Language + MSFsc + MSFsc:School timing	6	227.109	4.060	0.042	7.613	0.882
2	Math + Language + MSFsc + School timing + MSFsc:School timing	8	227.879	4.830	0.029	11.188	1.049
3	Math + Language + MSFsc + MSFsc:School timing + Math:School timing	8	227.413	4.363	0.036	8.860	0.947
4	Math + Language + MSFsc + MSFsc:School timing + Language:School timing	8	224.541	1.492	0.150	2.108	0.324
**5**	**Math + Language + MSFsc + MSFsc:School timing + Language:School timing + Math:MSFsc**	**9**	**223.050**	**0.000**	**0.322**	**1.000**	**0.000**
6	Math + Language + MSFsc + MSFsc:School timing + Language:School timing + Math:MSFsc + Language:MSFsc	10	225.021	1.972	0.118	2.680	0.428
7	Math + Language + MSFsc + MSFsc:School timing + Language:School timing + Math:MSFsc + Math:Language	10	224.519	1.470	0.152	2.085	0.319
8	Math + Language + MSFsc + MSFsc:School timing + Language:School timing + Math:MSFsc + Language:School timing:MSFsc	12	229.142	6.093	0.015	21.037	1.323
9	Math + Language + MSFsc + MSFsc:School timing + Language:School timing + Math:MSFsc + Math:MSFsc:School timing	11	227.063	4.014	0.043	7.440	0.872
10	Math + Language + MSFsc + MSFsc:School timing + Language:School timing + Math:MSFsc + Math:Language:MSFsc:School timing	12	227.879	4.829	0.029	11.103	1.045

Supplementary Table 8. ANOVA table for the model explaining grade retention. Both chronotype and grades are added as numerical variables in this model and school timing as a factor of 3 levels. Factors were added sequentially in the order presented here and a one-sided chi-squared test was performed to evaluate the significance of the deviance explained by each factor. n = 407.

	d.f	Deviance	Residual Deviance	P	Partial η2	90% CI
Math grades	1	51.156	236.013	<0.0001	0.130	[0.083-0.182]
Language grades	1	18.006	218.006	<0.0001	0.046	[0.018-0.084]
MSFsc	1	3.050	214.957	0.081	0.007	[0.000-0.027]
School timing:Language grades	2	0.406	214.550	0.816	0.002	[0.000-0.011]
MSFsc:Math grades	1	2.695	211.856	0.101	0.029	[0.008-0.062]
School timing:MSFsc	2	7.260	204.596	0.027	0.016	[0.000-0.040]

Supplementary Table 9. Summary of the model explaining grade retention. As models are logistic regression models, the slopes (calculated from Betas) obtained from them would allow calculation of the odd ratios of experience grade retention associated with each explanatory variable. Two-sided z-test were performed to test the significance of each beta. Both chronotype and grades are added as numerical variables in this model and school timing as a factor of 3 levels. Reference level: Morning school timing. n = 407.

		Beta	95% CI	z-value	p
1	Intercept	-1.776	[-7.411 to 4.144]	-0.609	0.542
2	MSFsc	1.394	[0.397 to 2.379]	2.779	0.005
3	Math grades	0.587	[-0.557 to 1.614]	1.074	0.283
4	Language grades	-1.134	[-1.751 to -0.612]	-3.952	<0.0001
5	Afternoon:Language grades	0.440	[-0.134 to 1.070]	1.464	0.143
6	Evening:Language grades	0.694	[0.200 to 1.277]	2.585	0.010
7	MSFsc:Math grades	-0.173	[-0.343 to 0.006]	-1.959	0.050
8	Afternoon:MSFsc	-0.349	[-0.854 to 0.130]	-1.409	0.159
9	Evening:MSFsc	-0.556	[-1.016 to -0.147]	-2.549	0.011

Supplementary Table 10. Mean sleep timings, sleep duration and social jetlag and sleep loss of students attending morning, afternoon or evening school timings. Some of this information was previously published in Rodríguez Ferrante et al. 2022[1]. M: mean. SD: standard deviation. N=259.

		**Morning**				**Afternoon**				**Evening**			
		**1st year**		**5th year**		**1st year**		**5th year**		**1st year**		**5th year**	
		**M**	**SD**	**M**	**SD**	**M**	**SD**	**M**	**SD**	**M**	**SD**	**M**	**SD**
**Week days**	**Sleep onset (SOnW)**	23:39	01:09	00:38	01:14	00:05	01:08	01:30	01:13	00:44	00:55	01:55	01:13
	**Sleep offset (SOffW)**	06:19	00:22	06:25	00:30	08:31	01:02	09:12	01:16	09:15	01:21	09:58	01:13
	**Sleep duration (SDw)**	6.672	1.152	5.788	1.158	8.430	1.354	7.699	1.480	8.526	1.339	8.034	1.422
**Free days**	**Sleep onset (SOnF)**	02:04	01:43	02:22	01:23	01:46	01:30	03:08	01:20	02:15	01:16	03:21	01:25
	**Sleep offset (SOffF)**	11:18	01:47	11:30	01:41	11:10	01:32	11:59	01:31	11:05	01:37	12:11	01:32
	**Sleep duration (SDf)**	9.231	1.405	9.145	1.390	9.400	1.419	8.863	1.389	8.845	1.536	8.834	1.218
	**Sleep loss (SDf-SDw)**	2.560	1.818	3.357	1.791	0.970	1.536	1.165	1.749	0.319	1.724	0.800	1.669
	**SJL**	3.696	1.269	3.403	1.228	2.165	1.131	2.203	0.959	1.678	1.104	1.819	0.979
	**MSFsc**	05:46	01:27	05:44	01:13	06:07	01:22	07:08	01:19	06:33	01:23	07:29	01:24

Supplementary Discussion

The results presented here show some differences with previous results from our own group[2]. The main difference is that in our previous study we reported a positive slope describing the association between grades and chronotype, i.e. an association between higher language grades and later chronotypes, which significantly differed from the negative slopes found for morning and afternoon school timings. In contrast, here the 5th year language grades' slopes do not differ between school timings and are all negative. This difference may be, at least, partially explained by the fact that 5th year students that participated in the previous study exhibited considerably later chronotypes (by 32 min) than those participating here (08:01 (SD=1:46) vs. 07:29 (1:24), respectively). Notably neither slope corresponding to the evening school schedule, in this study or in the previous one, were significantly different from zero. Another difference is that the magnitude of the association between late chronotypes and lower math grades for morning-attending students in 5th year reported here is stronger than those reported previously, not only in Argentina by our group[2] but also in other countries[1,3–6]. When comparing our results with those of other countries the difference is not surprising, considering that Argentinian students

present particularly late chronotypes[3,7–12] which, as we stated before, puts them in an especially susceptible situation when attending school in the morning. However, it is not clear why we observed such a difference between two populations of Argentinian students of the same age, from the same school and with similar schedules[2]. In particular, we are not aware about any changes associated with school or even social or cultural variables between the corresponding data collection years (i.e. 2015 and 2019) that could explain this difference. Interestingly, the magnitude of the slope reported here highlights how big the disadvantage of morning-attending students with later chronotypes can be compared to those with earlier chronotypes. To illustrate, consider two hypothetical 5th year students attending school in the morning, one whose chronotype is in the earlier quartile and the other in the later quartile. The one with the earlier chronotype would likely have math grades 0.8/10 point higher than the other. This difference in grades would double, exceeding 1.6 points, when comparing two other students, each of them from one of the most extreme chronotype deciles. This could lead not only to inequalities in their academic achievements and future income[13,14], but also to a gap in the adolescents' subjective well being[15,16]. Besides the mentioned differences, most results presented here and in our previous study[2] were consistent between both studies reinforcing our conclusion that both there exist a Chronotype and a Synchrony effect modulating academic performance.

References

1. Rodríguez Ferrante, G., Goldin, A. P. & Leone, M. J. School timingsThe Perfect Hurricane in Latin America: School Start Time, Chronotype, Sleep, and Academic Performance During Adolescence. in *Cognitive Sciences and Education in Non-WEIRD Populations: A Latin American Perspective*

(eds. Alves, M. V., Ekuni, R., Hermida, M. J. & Valle-Lisboa, J.) 207–226 (Springer International Publishing, 2022). doi:10.1007/978-3-031-06908-6_13.

2. Goldin, A. P., Sigman, M., Braier, G., Golombek, D. A. & Leone, M. J. Interplay of chronotype and school timing predicts school performance. *Nat. Hum. Behav.* 1–10 (2020) doi:10.1038/s41562-020-0820-2.
3. Preckel, F. *et al.* Morningness-eveningness and educational outcomes: the lark has an advantage over the owl at high school. *Br. J. Educ. Psychol.* **83**, 114–134 (2013).
4. Zerbini, G. *et al.* Lower school performance in late chronotypes: underlying factors and mechanisms. *Sci. Rep.* **7**, 4385 (2017).
5. Estevan, I., Silva, A. & Tassino, B. School start times matter, eveningness does not. *Chronobiol. Int.* **35**, 1753–1757 (2018).
6. Kolomeichuk, S. N., Randler, C., Shabalina, I., Fradkova, L. & Borisenkov, M. The influence of chronotype on the academic achievement of children and adolescents – evidence from Russian Karelia. *Biol. Rhythm Res.* **47**, 873–883 (2016).
7. Randler, C., Faßl, C. & Kalb, N. From Lark to Owl: developmental changes in morningness-eveningness from new-borns to early adulthood. *Sci. Rep.* **7**, (2017).
8. Roenneberg, T. *et al.* A marker for the end of adolescence. *Curr. Biol.* **14**, R1038–R1039 (2004).
9. Zerbini, G. & Merrow, M. Time to learn: How chronotype impacts education. *PsyCh J.* **6**, 263–276 (2017).
10. Dewald, J. F., Meijer, A. M., Oort, F. J., Kerkhof, G. A. & Bögels, S. M. The influence of sleep quality, sleep duration and sleepiness on school performance in children and adolescents: A meta-analytic review. *Sleep Med. Rev.* **14**, 179–189 (2010).
11. Han, C. H. & Chung, J. Late Chronotype is Associated with Adolescent Asthma: Assessment Using the Korean-Version MCTQ. *Int. J. Environ. Res. Public. Health* **17**, 3000 (2020).

12. Borisenkov, M. F., Perminova, E. V. & Kosova, A. L. Chronotype, Sleep Length, and School Achievement of 11- to 23-Year-Old Students in Northern European Russia. *Chronobiol. Int.* **27**, 1259–1270 (2010).

13. French, M. T., Homer, J. F., Popovici, I. & Robins, P. K. What You Do in High School Matters: High School GPA, Educational Attainment, and Labor Market Earnings as a Young Adult. *East. Econ. J.* **41**, 370–386 (2015).

14. Geiser, S. & Santelices, M. V. *Validity of High-School Grades in Predicting Student Success beyond the Freshman Year: High-School Record vs. Standardized Tests as Indicators of Four-Year College Outcomes. Research & Occasional Paper Series: CSHE.6.07*. *Center for Studies in Higher Education* (Center for Studies in Higher Education, 2007).

15. Crede, J., Wirthwein, L., McElvany, N. & Steinmayr, R. Adolescents' academic achievement and life satisfaction: the role of parents' education. *Front. Psychol.* **6**, (2015).

16. Bücker, S., Nuraydin, S., Simonsmeier, B. A., Schneider, M. & Luhmann, M. Subjective well-being and academic achievement: A meta-analysis. *J. Res. Personal.* **74**, 83–94 (2018).
