# Supplementary material for: A better alignment between chronotype and school timing is associated with lower grade retention in adolescents
Source: NPJ Sci Learn. 2023 Jun 21;8:21. doi: 10.1038/s41539-023-00171-0 (PMC10284813; doi:10.1038/s41539-023-00171-0)
Supplement: Supplementary file 2 — Reporting Summary [file 41539_2023_171_MOESM2_ESM.pdf]

nature portfolio

Corresponding author(s): Guadalupe Rodríguez Ferrante
Last updated by author(s): May 24, 2023

Reporting Summary

Nature Portfolio wishes to improve the reproducibility of the work that we publish. This form provides structure for consistency and transparency in reporting. For further information on Nature Portfolio policies, see our Editorial Policies and the Editorial Policy Checklist.

Statistics

For all statistical analyses, confirm that the following items are present in the figure legend, table legend, main text, or Methods section.

n/a	Confirmed	
☐	☒	The exact sample size (*n*) for each experimental group/condition, given as a discrete number and unit of measurement
☐	☒	A statement on whether measurements were taken from distinct samples or whether the same sample was measured repeatedly
☐	☒	The statistical test(s) used AND whether they are one- or two-sided<br>*Only common tests should be described solely by name; describe more complex techniques in the Methods section.*
☐	☒	A description of all covariates tested
☐	☒	A description of any assumptions or corrections, such as tests of normality and adjustment for multiple comparisons
☐	☒	A full description of the statistical parameters including central tendency (e.g. means) or other basic estimates (e.g. regression coefficient) AND variation (e.g. standard deviation) or associated estimates of uncertainty (e.g. confidence intervals)
☐	☒	For null hypothesis testing, the test statistic (e.g. *F*, *t*, *r*) with confidence intervals, effect sizes, degrees of freedom and *P* value noted<br>*Give P values as exact values whenever suitable.*
☒	☐	For Bayesian analysis, information on the choice of priors and Markov chain Monte Carlo settings
☐	☒	For hierarchical and complex designs, identification of the appropriate level for tests and full reporting of outcomes
☐	☒	Estimates of effect sizes (e.g. Cohen's *d*, Pearson's *r*), indicating how they were calculated

Our web collection on statistics for biologists contains articles on many of the points above.

Software and code

Policy information about availability of computer code

Data collection	No software was used
Data analysis	Data were analyzed using R system for statistical computing (v.4.0.2; R Core Team, 2020)

For manuscripts utilizing custom algorithms or software that are central to the research but not yet described in published literature, software must be made available to editors and reviewers. We strongly encourage code deposition in a community repository (e.g. GitHub). See the Nature Portfolio guidelines for submitting code & software for further information.

Data

Policy information about availability of data

All manuscripts must include a data availability statement. This statement should provide the following information, where applicable:

- Accession codes, unique identifiers, or web links for publicly available datasets
- A description of any restrictions on data availability
- For clinical datasets or third party data, please ensure that the statement adheres to our policy

The data and code that support the findings of this study are available from the corresponding author on request

Field-specific reporting

Please select the one below that is the best fit for your research. If you are not sure, read the appropriate sections before making your selection.

☐ Life sciences ☒ Behavioural & social sciences ☐ Ecological, evolutionary & environmental sciences

For a reference copy of the document with all sections, see nature.com/documents/nr-reporting-summary-flat.pdf

Behavioural & social sciences study design

All studies must disclose on these points even when the disclosure is negative.

Study description	Quantitative experimental, longitudinal study
Research sample	The sample are 407 students who completed a questionnaire in their 1st (june 2015) and a subsample of them who, again, complete the questionnaire in their 5th year (july 2019) of secondary school. The sample of students was balanced on gender (49.88% females of the 407 students; and 50.97% females from the 259 students) and it was age-homogeneous (1st year: M=13.49 y.o., SD=0.33 ; 5th year: M=17.58 y.o., SD=0.33).
Sampling strategy	The study was performed in two different moments (June 2015 and July 2019) at a local secondary school in the City of Buenos Aires, Argentina (34° 60′ S, 58° 38′ W). The school year starts in March and ends in December in Buenos Aires thus, the data was collected after three/four months of classes. All of the students who attended school on the corresponding day of data collection and were at 1st year (i.e. 2015) or at 5th year (i.e. 2019), were invited to participate in the study. Grades and students' lists where obtained from school authorities at the end of each academic year.
Data collection	During the typical hours of each school timing, students filled Spanish versions of the Munich chronotype Questionnaire (MCTQ) and demographic information (birth date and self-defined gender) using pen and paper. Grades and students' lists where obtained from school registries.
Timing	Data collection was conducted in June 2015 and july 2019.
Data exclusions	From the 436 and 352 students who completed the questionnaire in their 1st and 5th year, respectively, 259 students were included in the analyses performed in this study. Only those students who participate on the study on both years, who maintained their original school timing and with complete data on both years were included in academic performance analysis. Regarding grade retention analysis students with complete data in 2015 were included.
Non-participation	Any student refused to participate
Randomization	A crucial aspect of our experimental setup is that in this school, the school timing is set by a lottery system. At 12-13 y.o., just before the beginning of their first high school year, students from this secondary school are assigned by lots to one of three school timing or school hours (Morning: 07:45-12:05 am; Afternoon: 12:40-05:00pm; Evening: 05:20-09:40pm). Students cannot change the assigned school timing except if they already have brothers or sisters in the school hours they prefer to attend (which in turn were also originally assigned randomly to a given school timing), and they remain in the assigned school timing until the end of high school, fifth year, 17-18 y.o.

Reporting for specific materials, systems and methods

We require information from authors about some types of materials, experimental systems and methods used in many studies. Here, indicate whether each material, system or method listed is relevant to your study. If you are not sure if a list item applies to your research, read the appropriate section before selecting a response.

Materials & experimental systems

n/a	Involved in the study	
☒	☐	Antibodies
☒	☐	Eukaryotic cell lines
☒	☐	Palaeontology and archaeology
☒	☐	Animals and other organisms
☐	☒	Human research participants
☒	☐	Clinical data
☒	☐	Dual use research of concern

Methods

n/a	Involved in the study	
☒	☐	ChIP-seq
☒	☐	Flow cytometry
☒	☐	MRI-based neuroimaging

Human research participants

Policy information about studies involving human research participants

Population characteristics	See above
Recruitment	All first and fifth year students that attended school during the data collection process were recruited.
Ethics oversight	Ethical Committee of the Universidad Nacional de Quilmes

Note that full information on the approval of the study protocol must also be provided in the manuscript.
